# Refining the Universal, School-Based OurFutures Mental Health Program to Be Trauma Informed, Gender and Sexuality Diversity Affirmative, and Adherent to Proportionate Universalism: Mixed Methods Participatory Design Process

**DOI:** 10.2196/54637

**Published:** 2024-08-21

**Authors:** Lucinda Grummitt, Sasha Bailey, Erin V Kelly, Louise Birrell, Lauren A Gardner, Jillian Halladay, Cath Chapman, Jack L Andrews, Katrina E Champion, Emily Hunter, Lyra Egan, Chloe Conroy, Raaya Tiko, An Nguyen, Maree Teesson, Nicola C Newton, Emma L Barrett

**Affiliations:** 1 The Matilda Centre for Research in Mental Health and Substance Use The University of Sydney Sydney Australia; 2 School of Nursing McMaster University Ontario, ON Canada; 3 Peter Boris Centre for Addictions Research St Joseph's Healthcare Hamilton McMaster University Ontario, ON Canada

**Keywords:** mental health, prevention, school, depression, anxiety, proportionate universalism

## Abstract

**Background:**

Mental disorders are the leading cause of disease burden among youth. Effective prevention of mental disorders during adolescence is a critical public health strategy to reduce both individual and societal harms. Schools are an important setting for prevention; however, existing universal school-based mental health interventions have shown null, and occasionally iatrogenic, effects in preventing symptoms of common disorders, such as depression and anxiety.

**Objective:**

This study aims to report the adaptation process of an established, universal, school-based prevention program for depression and anxiety, OurFutures Mental Health. Using a 4-stage process; triangulating quantitative, qualitative, and evidence syntheses; and centering the voices of young people, the revised program is trauma-informed; lesbian, gay, bisexual, transgender, nonbinary, queer, questioning, and otherwise gender and sexuality diverse (LGBTQA+) affirmative; relevant to contemporary youth; and designed to tailor intervention dosage to those who need it most (proportionate universalism).

**Methods:**

Program adaptation occurred from April 2022 to July 2023 and involved 4 stages. Stage 1 comprised mixed methods analysis of student evaluation data (n=762; mean age 13.5, SD 0.62 y), collected immediately after delivering the OurFutures Mental Health program in a previous trial. Stage 2 consisted of 3 focus groups with high school students (n=39); regular meetings with a purpose-built, 8-member LGBTQA+ youth advisory committee; and 2 individual semistructured, in-depth interviews with LGBTQA+ young people via Zoom (Zoom Video Communications) or WhatsApp (Meta) text message. Stage 3 involved a clinical psychologist providing an in-depth review of all program materials with the view of enhancing readability, improving utility, and normalizing emotions while retaining key cognitive behavioral therapy elements. Finally, stage 4 involved fortnightly consultations among researchers and clinicians on the intervention adaptation, drawing on the latest evidence from existing literature in school-based prevention interventions, trauma-informed practice, and adolescent mental health.

**Results:**

Drawing on feedback from youth, clinical psychologists, and expert youth mental health researchers, sourced from stages 1 to 4, a series of adaptations were made to the storylines, characters, and delivery of therapeutic content contained in the weekly manualized program content, classroom activities, and weekly student and teacher lesson summaries.

**Conclusions:**

The updated OurFutures Mental Health program is a trauma-informed, LBGTQA+ affirmative program aligned with the principles of proportionate universalism. The program adaptation responds to recent mixed findings on universal school-based mental health prevention programs, which include null, small beneficial, and small iatrogenic effects. The efficacy of the refined OurFutures Mental Health program is currently being tested through a cluster randomized controlled trial with up to 1400 students in 14 schools across Australia. It is hoped that the refined program will advance the current stalemate in universal school-based prevention of common mental disorders and ultimately improve the mental health and well-being of young people in schools.

## Introduction

### Background

Mental disorders, such as depression and anxiety, are the leading causes of disease burden among youth [1]. Almost two-thirds of those who experience a mental disorder are first diagnosed before the age of 25 years [2,3]. Global evidence suggests depressive and anxiety symptoms have been increasing among adolescents since the 1980s [4-7]. This was further compounded by the COVID-19 pandemic, culminating in a current crisis characterized by doubled prepandemic rates of depression and anxiety among youth and increased attempts at suicide [8,9]. These trends highlight the urgency of prioritizing efforts to address mental ill-health.

Alarmingly, many people experiencing mental health disorders do not receive the care they need. In high-income countries, it is estimated that only 33% of people with depression access treatment, while in low-income countries, just 8% of those in need access treatment [10]. Services are overburdened, with high out-of-pocket costs, lengthy wait times, and other barriers prohibiting access to treatment for our youth [11]. While improving access to ensure treatment coverage for those who need it is critical, greater attention and resources must also be directed to *preventing* these disorders.

Schools are an ideal setting for implementing mental health prevention programs [12], enabling broad reach before the peak age of onset of mental disorders (14.5 years [2]). Universal delivery of mental health programs is typically low cost and practical for schools, enables tailoring of programs to students’ developmental level, facilitates scalability, and is nonstigmatizing [13,14]. However, meta-analyses and recent large-scale randomized controlled trials (RCTs) of universal school-based depression and anxiety prevention programs show mixed results, including null effects, small benefits on symptom reduction in the short term, and even potential iatrogenic effects, particularly for individuals with preexisting mental health symptoms [15-20]. Indeed, the consistent observation of small, null, and iatrogenic effects of universal school-based mental health prevention activities has garnered significant global attention [21-23], with several editorials calling for a shift in universal approaches for school-based prevention for depression [21,23].

Therefore, despite the promise of school-based, universal mental health prevention, existing evidence demonstrates a clear need to improve these interventions or rethink the approach used in universal school-based prevention. One solution that has been proposed is the use of targeted prevention approaches, which selectively target subgroups within the population that have been identified as being at an elevated risk for mental ill-health [12]. Meta-analytic estimates of targeted school-based depression and anxiety prevention programs consistently show larger effect sizes compared with universal programs [16,17]. However, targeted programs often incur greater implementation and resource burden, requiring sound identification and communication of individual risk, both in terms of sensitivity and specificity in allocating individuals to receive an intervention or not, as well as care to avoid labeling or causing stigma through this process [24].

One potential path forward that leverages the strengths of both universal and selective prevention is “proportionate universalism.” This is the notion that universal public health policies or interventions be designed to proportionately (equitably) benefit those who need it the most [25,26]. A growing body of public health and health policy literature suggests that this is a pragmatic step toward achieving optimal global health by harnessing universal delivery but tailoring intervention dosage and content based on need [25]. By applying proportionate universalism to mental health prevention, those exhibiting elevated mental health symptomatology would receive or could opt in to receive additional intervention components, such as psychoeducation, skills training, and evidence–based early intervention resources. This has the potential to address a notable challenge in universal mental health prevention, whereby the same intervention is delivered to individuals with varying levels of preexisting symptoms and risk. For those with low symptoms or levels of risk, universal intervention may lack relevance and perceived usefulness, limiting engagement, while for those with preexisting symptoms, its low-intensity therapeutic content may raise awareness or discomfort of unpleasant thoughts and emotions but could be insufficient in helping these individuals manage their distress [20,27]. Providing flexibility in the dosage of the intervention, where individuals can choose to receive additional skills training or psychoeducation, could improve the relevancy of prevention SMS text messaging across the spectrum of preexisting symptomatology. Moreover, giving individuals a choice as to which intervention components they receive could improve adherence, retention, and ultimately, effectiveness [28]. In practice, however, literature is scant on real-life applications of this paradigm.

Another noticeable limitation of universal mental health prevention programs is a lack of attention to the social factors contributing to mental health difficulties. A wealth of literature has identified the elevated risk of mental health difficulties among young people exposed to trauma or adversity, such as child abuse, neglect, or exposure to domestic violence. Given that, up to 62% of Australians have experienced maltreatment or domestic violence during childhood and adolescence [29], programs aiming to address or prevent adolescent mental health problems must be trauma-informed. In addition, growing research has highlighted the disproportionate rates of mental ill-health among lesbian, gay, bisexual, transgender, nonbinary, queer, questioning, and otherwise gender and sexuality diverse (LGBTQA+) young people [30,31]. Nearly half of young LGBTQA+ Australians have ever received a diagnosis of generalized anxiety disorder and depression [32]. Transgender people and gender diverse people (binary and nonbinary; henceforth, “trans”) are people with a gender identity different from the one presumed for them at birth. Relative to their sexuality diverse peers, transgender young people report even higher rates of generalized anxiety disorder and depression conditions [32]. Mental ill-health disparities affecting LGBTQA+ young people widen through adolescence [33]. LGBTQA+ young people experience daily, chronic discrimination based on their genders and sexualities and, moreover, are more likely than their cisgender, heterosexual peers to report nonidentity–related traumatic events, such as intimate partner abuse and sexual assault [34]. Notably, it is those experiences of daily, chronic discrimination and inability to feel safe that bear more mental health consequences than traumatic events [34]. Moreover, the mental health effects of both minority stressors and nonidentity–specific traumatic events are disproportionately shouldered by transgender young people [34]. Although schools can be affirming and supportive of LGBTQA+ young people [35], high rates of school-based bullying, harassment, exclusion, and isolation continue to be documented among contemporary LGBTQA+ young people [36]. Significant variability in school and staff-level preparedness to support underscores a dire need for additional school-wide LGBTQA+ affirmative initiatives [30]. Henceforth, to effectively respond to either the needs of young people exposed to trauma or adversity or LGBTQA+ young people necessitates an integrated approach, reflecting how young people undergo dynamic, interrelated experiences of trauma, adversity, gender, and sexuality. Therefore, a trauma-informed, LGBTQA+ affirmative approach that applies proportionate universalism may be effective in preventing and reducing the escalation of mental health symptoms among adolescents, particularly adolescents exposed to trauma or adversity and LGBTQA+ adolescents.

One universal, school-based OurFutures Mental Health program (formerly Climate Schools Mental Health) has similarly shown mixed effects for preventing anxiety and depression [37]. It was developed in 2009 as a web-based cognitive behavioral therapy (CBT)–based mental health education program. The content was delivered over six 30-minute lessons (including a 10-minute weekly revision) comprising skill acquisition; psychoeducation; and management of thoughts, emotions, and behaviors specific to depression and anxiety [38]. A feasibility trial demonstrated improvements in well-being and knowledge about stress and reductions in measures of distress [38]. The program was subsequently divided into 2 programs targeting depression and anxiety separately and was tested in a 3-arm, cluster RCT with 976 year 9 and 10 students from 12 Australian schools. Compared with health education as usual, the anxiety intervention resulted in significant reductions in anxiety symptoms, and the depression intervention reduced both anxiety and depressive symptoms [39].

In 2013, the depression and anxiety modules were amalgamated and condensed into six 40-minute lessons to create the Climate Schools Mental Health program to reduce content overlap and decrease time demands for schools [40]. Lessons included a 20-minute cartoon storyline, followed by optional teacher–delivered activities involving CBT–based skills practice. Teachers and students were also provided with downloadable lesson summaries to reinforce the content delivered in the lessons. The program was developed through input from an experienced clinical psychologist; focus groups with approximately 30 students from 2 independent schools in Sydney, Australia; and consultation with numerous health and educational professionals to verify the program’s clinical and educational validity [40]. Between 2013 and 2016, a cluster RCT of >6000 students of grades 8 and 9 (aged approximately 13-15 years) investigated the effectiveness of 3 interventions, including Climate Schools Mental Health, Climate Schools Substance Use, and both Climate Schools Mental Health and Climate Schools Substance Use programs combined, for preventing depression, anxiety, and substance use, accordingly, compared with an active control (health education as usual). Additional detail regarding the components of Climate Schools Substance Use is published elsewhere [41]. Over a 30-month period, students in the combined group experienced increased knowledge of alcohol, cannabis, and mental health and decreased growth in anxiety symptoms [42]. However, further post hoc analyses revealed the students in the stand-alone Climate Schools Mental Health condition improved their knowledge of mental health but reported no improvement in other mental health outcomes compared with the control condition [37]. Moreover, the participants in the stand-alone mental health group also reported transient increased internalizing scores at the 6-month and 12-month postintervention time points compared with controls; however, these were not sustained at further follow-ups [37].

### Objectives

In light of previous findings from RCTs of the OurFutures Mental Health program, as well as existing evidence from other universal school-based mental health prevention approaches, the OurFutures Mental Health program was refined from 2022 to 2023. This paper describes this refining process. We aimed to explore and understand young peoples’ perceptions and experiences of the OurFutures Mental Health program within previous trials, source input from young people to refine the program to be trauma-informed and gender and sexuality inclusive, and incorporate the principles of proportionate universalism.

## Methods

### Overview

Program adaptation occurred from April 2022 to July 2023, using a four-stage approach: (1) analysis of evaluation data from the original Climate Schools Mental Health program; (2) focus groups and interviews conducted with young people, including LGBTQA+ youth; (3) content review by a clinical psychologist; and (4) expert team consultation and literature review.

This paper adheres to guidance for reporting intervention development studies in health research (Guidance for the Reporting of Intervention Development) [43].

### Ethical Considerations

Ethics approval was granted by the University of Sydney Human Research Ethics Committee (2022/664) and for Stage 1 from the University of New South Wales Human Research Ethics Committee (HC13073). All participants provided written informed consent. In stage 1, participants were entered into a prize draw to win an iPad. In stage 2, focus group participants were entered into a prize draw to win an Aus $100 (US $65.84) gift card, and interview participants were reimbursed with an Aus $20 (US $13.17) gift card.

### Stage 1: Analysis of Evaluation Data From the Original Climate Schools Mental Health Program

Between June 2022 and February 2023, we conducted a mixed methods analysis of student evaluation data (n=762, mean age 13.5, SD 0.62 y) collected immediately after the delivery of the Climate Schools Mental Health program (2015) in a previous trial. The full methodology of the trial is published elsewhere [42,44]. Students responded to seven 5-point Likert scale items regarding how strongly they agreed or disagreed with statements regarding satisfaction, relevance, and helpfulness of the program. Two open-ended items also asked students to identify “one good thing” and “one bad thing” about the program. Open-ended items were analyzed using a 6-step thematic analysis approach [45]. Differences in responses by sex and baseline mental ill-health symptoms (Generalized Anxiety Disorder questionnaire [46], Patient Health Questionnaire-9 [47], and Emotional Symptoms subscale of the Strengths and Difficulties Questionnaire [48]) were also tested using multivariate logistic regression models, which controlled for binary sex. Full information on the evaluation has been published elsewhere [27].

### Stage 2: Focus Groups and Interviews Conducted With Young People, Including LGBTQA+ Youth

#### Overview

The adaptation involved extensive consultation with young people, including LGBTQA+ young people. Duty of care procedures were created, including having a clinical psychologist on standby, although these procedures were not required during the consultations. Specifically, this consultation process comprised 3 stages.

#### Stage 2.1: School-Based Focus Groups (General Population of Adolescents)

To seek input on current sources of stress experienced by young people, as well as feedback on the OurFutures Mental Health storylines, focus groups were conducted in 2 Australian high schools. Given that 1 school had a large student cohort, 2 separate focus groups were conducted within the school, resulting in 3 focus groups across 2 schools (n=39). It was determined that this is an appropriate number for data saturation [49,50].

Independent secondary schools in Sydney, Australia, were contacted in mid-2022 using publicly available email addresses and phone numbers and invited to participate in focus groups. Two schools agreed to participate and sent information and consent forms to parents of their grade 8 students (aged approximately 13-14 years). Students were required to sign their own information and consent form.

Focus groups were each cofacilitated by 2 researchers each and took place in November 2022 and February 2023. A semistructured focus group guide was used, comprising 12 questions (Multimedia Appendix 1) centered on major stressors, how they affect mental health, and strategies youth use to cope. Students were also shown scenes from the existing program, and facilitators sought specific feedback regarding the characters, storylines, and language. This set of discussion questions permitted flexibility for participants to expand on their responses and explore themes in greater depth.

School staff were not present in the room during the focus groups. Focus groups were audio-recorded and later transcribed using transcription software Otter (Otter.ai, Inc). Focus groups lasted for approximately 40 minutes, and participants could opt in to enter a draw to receive an Aus $100 (US $65.84) gift voucher (1 per school).

#### Stage 2.2: Consultations With an LGBTQA+ Youth Advisory Committee

An LGBTQA+ youth advisory committee (YAC) was established, comprising 8 LGBTQA+ young people aged 17 to 19 years across rural or regional and metropolitan areas in New South Wales, Australia. There was a diverse representation of gender and sexuality in the YAC membership. YAC members were recruited through advertisements shared through professional networks, including newsletters and social media channels. Targeted advertisements were further shared through collaborative efforts from local LGBTQA+ youth health organizations. LGBTQA+ YAC members were reimbursed with Aus $30 (US $19.75) gift vouchers for each meeting they attended. Over the course of intervention adaptation, the YAC met 3 times: August 2022, November 2022, and March 2023. Chaired by SB, these early meetings focused on high-level discussions about the process and ethics of adapting universal programs intended to reach all young people, to be simultaneously affirming, inclusive, and supportive of LGBTQA+ young people. In the interest of maintaining comfortable, confidential spaces, these meetings were not recorded; however, detailed independent field notes were taken by SB and RT for later comparison and review. Iterative discussion based on these field notes generated guiding principles and accompanying recommendations stemming from LGBTQA+ YAC discussions for the adaptation of OurFutures Mental Health to be LGBTQA+ affirming and inclusive.

#### Stage 2.3: Interviews With LGBTQA+ Youths

LGBTQA+ young people aged 16 to 21 years were invited to participate in individual, semistructured, in-depth interviews. Interview questions (Multimedia Appendix 1) were structured similarly to focus group items and aimed to explore 2 main domains: contemporary sources of stress experienced by LGBTQA+ young people and potential areas for making the OurFutures Mental Health program more affirming, inclusive, and supportive of LGBTQA+ young people. Young people were recruited through social media (Twitter; Twitter, Inc, subsequently rebranded as X and Facebook; Meta Platforms, Inc) and professional networks of youth and LGBTQA+ organizations. Interviews were conducted over Zoom or WhatsApp text messaging (participant’s choice) and lasted for approximately 1 hour. These online channels were chosen as they offer LGBTQA+ young people increased flexibility, confidentiality, privacy, and anonymity to overcome key barriers to participation, including travel, cost, and parental support [51,52]. Interview participants were reimbursed with an Aus $20 (US $13.17) for their time. Interview facilitators had a script but had the flexibility to explore participant responses in depth and ask follow-up questions based on participant responses. Interviews were recorded and transcribed, and summative content analysis was conducted.

### Stage 3: Clinical Psychologist Review of All Program Materials

From April to July 2023, a clinical psychologist provided an in-depth review of all program material to enhance readability (condensing text and simplifying language); improve utility (increasing use of role modeling techniques by characters) while preserving key CBT elements; and remove focus on overt symptoms and disorders, instead normalizing general stress and worry or feeling low; and differentiating between the universal experience of emotions and mental disorders, such as anxiety and depressive disorders [22]. The psychologist review also incorporated considerations of safety and privacy, suggestions for trauma-informed content, and reviewing trauma-informed refinements (refer to the Stage 4: Expert Team Consultation and Literature Review section).

### Stage 4: Expert Team Consultation and Literature Review

From April 2022 to May 2023, a team of 12 research experts in adolescent mental health and a clinical psychologist engaged in fortnightly consultations on the intervention adaptation. Aligning with contemporary discussions regarding the future of universal, school-based mental health prevention programs, the team conscientiously discussed potential strategies to avoid potential iatrogenic harms associated with the program [21], including normalizing difficult emotions, discussing character examples rather than personal examples, and offering strategies for personal reflection and skills practice as optional and for discretionary individual use out of class. Discussions focused on the latest literature and findings from school-based universal mental health trials [19,23,37,40], as reviewed in the introduction of this study. These consultations drew on the latest evidence for trauma-informed prevention [53-55]. Specifically, these drew on 2 systematic reviews of the literature, which identified intervention targets to prevent psychopathology for young people exposed to trauma, as well as existing trauma-informed mental health prevention programs for young people aged 12 to 24 years [56,57].

Finally, consultations centered on restructuring the existing program and adapting it in line with the youth feedback described in stages 1 and 2.

Formative findings and insights from the aforementioned stages were collated and iteratively discussed among the research team. Adaptations were subsequently made to the OurFutures Mental Health intervention relating to the delivery of therapeutic content (classroom materials and take-home activities), storylines, and cartoons.

## Results

### Stage 1: Analysis of Evaluation Data From the Original Climate Schools Mental Health Program

Textbox 1 presents the identified areas for improvement arising from the formative evaluation and the corresponding intervention adaptation undertaken. Full results of the formative evaluation are presented in a separate publication [27]. Briefly, while most (453/759, 59.7%) students rated the OurFutures Mental Health program as good or very good, a considerable proportion of students did not perceive the original storylines to be relevant and were unsure whether they were likely to use skills and information from the program in their own lives (370/758, 48.8% and 290/757, 38.3%, respectively). Female participants were less likely to perceive the storyline as relevant compared with their male peers. Adjusting for participants’ sex, participants with either probable anxiety disorder, probable depressive disorder, or elevated levels of emotional symptoms were significantly more likely to enjoy the project storylines compared with their peers without these 3 respective conditions [27].

Five broad themes emerged from participants’ survey responses to open-ended questions regarding potential areas for improvement: (1) making content more realistic and relatable; (2) ensuring that the content is engaging and entertaining; (3) ensuring that the content covers wide aspects of mental ill-health, such as adversity, stigma, and diversity; 4) reducing the length of the program; and 5) ensuring that the materials are age appropriate.

**Areas for future improvement identified in previous program iteration.**

**Focus areas**

- The stories seemed unrealistic or unrelatable, for example, having cognitive behavioral therapy skills delivered by 1 of the peer characters in the cartoon (Chloe, a student aged 14 years).
- The content was not engaging and entertaining.
- The program did not adequately cover the realities of living with mental ill-health, including adversity, stigma, and diversity.
- There was too much information, and the program was too lengthy.
- The content seemed to be aimed at younger audiences.

### Stage 2.1: School-Based Focus Groups (General Population of Adolescents)

Three in-depth focus groups were conducted in 2 secondary schools in Australia, with youths aged 13 to 15 years (n=39). Predominant responses to the research questions eliciting the main causes of stress among Australian youths, ways of coping, and topics they believe should be covered in a mental health program are presented in Textbox 2 and Multimedia Appendix 1.

Some of the students’ comments vividly described scenarios reflective of contemporary causes of stress and coping strategies. For example, 1 participant noted that “lash[ing] out... is a very common experience.” Another focus group participant raised that “a lot of young people today report feeling rushed....” These discrete comments were addressed through specific changes and adaptations in the cartoon and learning materials. Other responses, which were more intangible and commentary in nature, were accordingly absorbed into the team’s general outlook and approach to the intervention adaptation process. For example, when describing the therapeutic experience of successfully coping with a difficult time, 1 focus group participant described it as “...doing things so you can feel like out of your reality.”

In addition to the common sources of stress mentioned above, focus group participants also identified discrete aspects of the OurFutures Mental Health cartoons and storylines that would benefit from improved relevance. These areas for improvement are listed below in Textbox 3.

**Focus group participants’ common sources of distress, ways of coping, and topics they believe should be covered in a school-based mental health program.**

**Research questions and responses**

- Concerns
  Bullying
  Gender and sexuality diversity
  School work and pressures, for example, deadlines, time management, managing cocurricular activities, and relationships on top of school
  Parent expectations
  Sport (eg, how well you perform and relationships with members of the team)
  Relationships, particularly peer relationships
- Ways of coping
  Try not to think about it
  Talking with friends
  Seeking help from a professional (eg, psychologist)
  Finding someone you can talk to who understands you
  Telling someone (instead of being mean back, eg, bullying)
  Being around supportive friends
  Social media and games
  “Breathing tricks to help”
  Taking a broader perspective
  Preparing the best you can
  Avoiding distractions
  Breaking things up with a run or seeing friends
  Letting things go
  Listening to each other
  Asking for advice
- Topics and issues
  Social media
  How to be assertive
  False information (eg, rumors), social media depictions, stereotypes, and stigma
  How to seek help from an adult
  Substance use, especially vapes
  Diverse representations of gender and sexuality
  Normalization of gender and sexuality diversity
  Combating transphobia and racism
  Trauma
  Time management
  Planning activities
  Important role of friendships
  Lack of support from friends
  How to have and “manage” healthy relationships with friends
  Mindset

**Focus group participants’ suggestions for improvement.**

**Suggestions**

- Remove curfew for characters as this is not realistic for young people, who are more likely to communicate with their parents via phone about where they are and what time they will be home.
- Need to increase engagement
- Trans and gay people in modules should “just be there” and not be different.
- Characters looked like Disney princesses. Clothes were “basic” and not realistic.
- Gender stereotypes of boys going to the beach and girls hanging out at home should be avoided.
- Inclusive, stigma-free language around mental health should be used.
- Animations appeared dated

### Stage 2.2: Consultations With an LGBTQA+ YAC

Insights drawn from YAC meetings and accompanying field notes were distilled into key recommendations that were adopted by the researchers in their approach and activities to ensure that all program content and materials are affirming, inclusive, and supportive of LGBTQA+ young people and gender and sexuality diversity more broadly. These guiding principles and key recommendations are detailed in Textbox 4.

**Guiding principles and recommendations for making universal mental health prevention programs inclusive, supportive, and affirming of lesbian, gay, bisexual, transgender, nonbinary, queer, questioning, and otherwise gender and sexuality diverse (LGBTQA+) young people, as described by the LGBTQA+ youth advisory committee.**

**Guiding principles and recommendations**

- LGBTQA+ young people experience life unrelated to their gender and sexuality.
  Produce narratives that do not center LGBTQA+ young peoples’ storylines around their gender and sexuality.
- Being inclusive of LGBTQA+ young people means letting LGBTQA+ young people “casually exist,” that is, “inclusion through normalisation.”
  Normalize gender and sexuality diversity for an LGBTQA+ character by not making gender and sexuality a focal part of their personality.
- Avoid tokenistic stereotypes but portray genuine “touchstones” of trans and queer cultures.
  Draw from real-life experiences to create authentic, relatable depictions of LGBTQA+ young people.
- Queer and transgender identities are diverse and intersectional.
  Ensure representation of less represented LGBTQA+ young people, including trans and nonbinary people, as well as LGBTQA+ young people at different stages of their identity (eg, questioning).
- Queer and transgender identities cannot be seen by other people unless you choose to express and “show” it.
  Ensure all storylines and characters reflect the complex nuance of gender and sexuality diversity rather than inauthentic, simplistic, or tokenistic expressions.

### Stage 2.3: Interviews With LGBTQA+ Youths

Two LGBTQA+ youths, a gay transgender boy and a bisexual cis girl, consented to participate in interviews via WhatsApp text messaging and Zoom videoconferencing, respectively. Interviews highlighted how isolation is a common issue for LGBTQA+ young people. Moreover, both participants reflected on how their families and friends represented major support systems. For example, 1 participant noted:

I have my support systems for when I feel unsafe but I think the majority of the time there’s only avoidance.

Both participants noted that their sexuality and gender were not causes of distress but rather prejudice and discrimination from others due to their sexuality and gender caused distress. In addition, both participants noted that the current materials did not make any mention or representation of gender and sexuality diversity, with 1 participant suggesting that “...seeing other queer people comfortable and thriving is all a lot of kids need.” Input from these interviews was combined with feedback from the YAC consultations (refer to the Stage 2.2: Consultations With an LGBTQA+ YAC section) to adapt the intervention accordingly.

### Stage 3: Clinical Psychologist Review of All Program Materials

The review by a clinical psychologist was driven by key guiding principles and accompanying recommendations, as shown in Textbox 5.

**Guiding principles and recommendations following clinical psychologist review of the program materials.**

**Guiding principles and recommendations**

- Difficult emotions are experienced by everyone and have a normal ebb and flow, just as pleasant emotions do. Emotions are informative.
  Avoid pathologizing difficult emotions.
  Acknowledge that while emotions can be unpleasant, they can also be informative.
  Promote student understanding of emotions by encouraging them to think of other words to describe their emotions, for example, the color wheel.
  Language such as “activities to combat depression” should be avoided. Provide coping strategies along with the knowledge that these are not intended to remove uncomfortable emotions but rather help tolerate when they are overwhelming.
- How we cope with our emotions and thoughts is important; this is the difference between low mood and depressive disorder and anxiety and anxiety disorder.
  Educate students on the difference between the symptoms and a disorder. Reinforce that all students experience difficult emotions, but not all students experience depression or anxiety.
  Provide strategies to promote healthy ways of coping when we inevitably experience periods of feeling anxious or down.
  Include behavioral activation strategies to promote functioning when feeling low or anxious.
- Stressful life events have a strong impact on the symptoms of depression and anxiety.
  Include psychoeducation, resources for teachers, and details of support services.
  Reduce the focus on the individual and educate students on the social determinants of health and trauma. This will hopefully reduce shame, stigma, and self-stigma.
- Adolescent depression often includes dysregulated emotions, labile mood and can include suicidality and self-harm [58].
  Provide guided examples to self-regulate in times of high emotions, such as breathing exercises, progressive muscle relaxation, and emotion surfing for all students and optional additional strategies, such as soothing activities and grounding techniques.
- There is a huge individual variability in strategies that young people find helpful for managing strong emotions [20,59,60]. Finding strategies that work for oneself enables empowerment and autonomy, which are key to adolescent development.
  Make all skills practice apply to the cartoon characters, with any self-applied skills training to be optional only. Students do not have to discuss and practice their own experiences in class (also recommended from a trauma-informed perspective, see Stage 4: Expert Team Consultation and Systematic Reviews) but can learn the skills through peers in the cartoon characters and then can choose to use the strategies in their own time.
  Rather than being prescriptive, phrasing refers to trying different strategies to see what helps them, and the characters use different types of strategies. This reinforces that not everything works for everyone and is hoped to reduce feelings of hopelessness if certain strategies do not help.
- Students have incredibly varied lived experiences, which influence their mental health in myriad ways.
  Scenarios used in class activities apply to the cartoon characters only. Students only apply skills to their own personal scenarios in the privacy of their own homes. This is hoped to reduce the negative impact of social comparison, whereby students who experience elevated depressive symptoms may feel more isolated and concerned whether the other students in the classroom reflect on their own experience of never having experienced elevated symptomatology.
- Sources of support are crucial
  Modeling characters seeking support
  Ask students to write down the name of someone who they could talk to about a problem they are currently facing, or if they cannot think of anyone, a service from one of those provided to them in the program that they could call. Identifying the barriers to reaching out to this person to talk to them about the problem they are facing and some things they could do to overcome this barrier

### Stage 4: Expert Team Consultation and Systematic Reviews

Program adaptation drew on the findings from 2 systematic reviews conducted from 2021 to 2023 and consultations among expert researchers and clinicians in the fields of childhood trauma and youth mental health. These systematic reviews identified intervention targets to prevent psychopathology for young people exposed to trauma, as well as existing trauma-informed mental health prevention programs for young people. Full details of these reviews are available elsewhere [56,57]. Guiding principles and recommendations derived from this literature and team consultations are presented in Textbox 6.

**Guiding principles and recommendations regarding embedding trauma-informed principles within the modified OurFutures Mental Health program generated from expert team consultation and systematic literature reviews.**

**Guiding principles and recommendations**

- Trauma-informed practice recognizes the impact, signs, and symptoms of trauma; resists retraumatizing clients or students; and integrates understanding of trauma into practice and policies [61].
  Traumatic events are commonly faced by young people and should be represented in program content. Psychoeducation around the impact of trauma on mental health and common signs and symptoms of trauma exposure should be included in accompanying student and teacher lesson summaries.
  School practices and policies play an important role in avoiding retraumatizing youth affected by trauma. For example, disciplinary practices that provide support rather than exclusion (eg, suspension) reflect trauma-informed practice.
  Provide information to teachers before program commencement via teacher summaries.
  All skills practice in the classroom apply to the cartoon characters. Students do not discuss their personal experiences or practice skills for their own situations in class, where they may not feel safe (due to peers and teachers). Instead, skills are learned through the cartoon characters, and students can choose to access the optional, at-home activities to apply the skills to their own scenarios.
- Many young people exposed to traumatic events do not develop mental health problems. Trauma should not be considered deterministic of mental illness. The individual and social resources available to someone exposed to trauma can affect the development of mental ill-health.
  Include information for teachers on the link between trauma and mental health and that it is not deterministic in the teacher summaries.
  Provide psychoeducation material to students, illustrating the interconnected relationship between thoughts, feelings, and actions.
- Exposure to adversity can impact one’s ability to regulate their own emotions.
  Empower students with skills of soothing, grounding techniques, imagery, and visualization techniques.
- There is a diverse range of exposures to adversity that impact young people differently.
  Represent a range of adversities in the program content, for example, bullying, domestic violence, and gender-based and sexuality-based discrimination.
  Similarly, represent a range of reactions, including feeling sad or down, anxious, and dysregulated.
- Safety is the first priority.
  Consistently encourage young people to talk to a trusted adult when faced with traumatic or adverse experiences. List examples of trusted adults to whom they can reach out, for example, a school counselor, teacher, parent, or other relative. Provide information and contact details for third-party support services.
- The impacts of trauma can be long term, affecting attribution styles, emotions, and behaviors. There are high levels of shame and self-criticism in adolescents, especially for those exposed to adversity and discrimination.
  Reflect on how experiences of past adversity can affect how we perceive ourselves, the world, and our relationships. Incorporate skills training and practice for students to identify thinking traps and challenge these with more helpful and realistic ways of thinking. Encourage self-compassion through student activities.

### Adapted OurFutures Mental Health Module

In response to the youth perspectives and guiding principles described in Textbox 6, the following adaptations were made to the OurFutures Mental Health program. Storyline dialogue and lesson content were reduced in length (quantity and overall time taken to complete).

#### Storylines

To reflect best-practice principles of trauma-informed approaches to prevention (Textbox 6), the research team carefully expanded 2 characters’ storylines, including Will and Chloe, to reflect the backgrounds of domestic violence and bullying, respectively, in an authentic, trauma-informed manner. These 2 specific experiences reflect the most commonly experienced adverse events by young people in Australia [62,63] and are strongly associated with mental ill-health and substance use [53,64,65]. In addition to these major changes, several minor modifications were made to the program storylines:

- Elements of drama in the storyline (eg, friendship conflicts and suspenseful romance) were increased, and dialogue was significantly shortened to increase student engagement.
- Several key expansions were made to characters’ storylines to include more diverse life experiences. These include themes of bullying, domestic violence, household dysfunction, and identity exploration. More diverse representations of depression and anxiety, incorporated issues of stigma and discrimination, the role of friendships, and the physiological aspects of anxiety were included.
- School stress and time management were made central and peripheral themes in all main characters’ storylines.
- Scenes were revised and café and skate park (gender-neutral) settings were introduced. Transgender nonbinary queer character, Julia, was included in both.
- Material was revised to remove curfews within characters’ storylines.
- Two new narrators were introduced to add more layers to the main characters’ respective storylines through focalizing narration from an outside character.
- Themes of peer relationships and intimate partner relationships were embedded and maintained throughout all character storylines.

#### Characters

**Figure 1:**
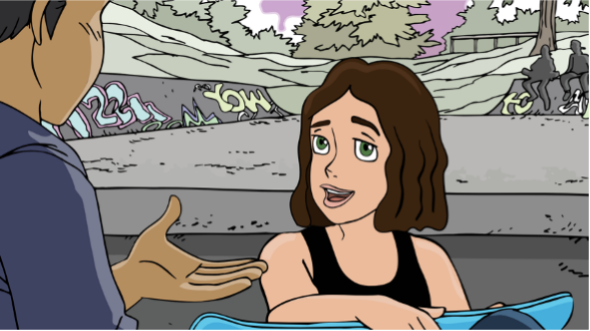
New character in the revised OurFutures Mental Health program: Julia.

**Figure 2:**
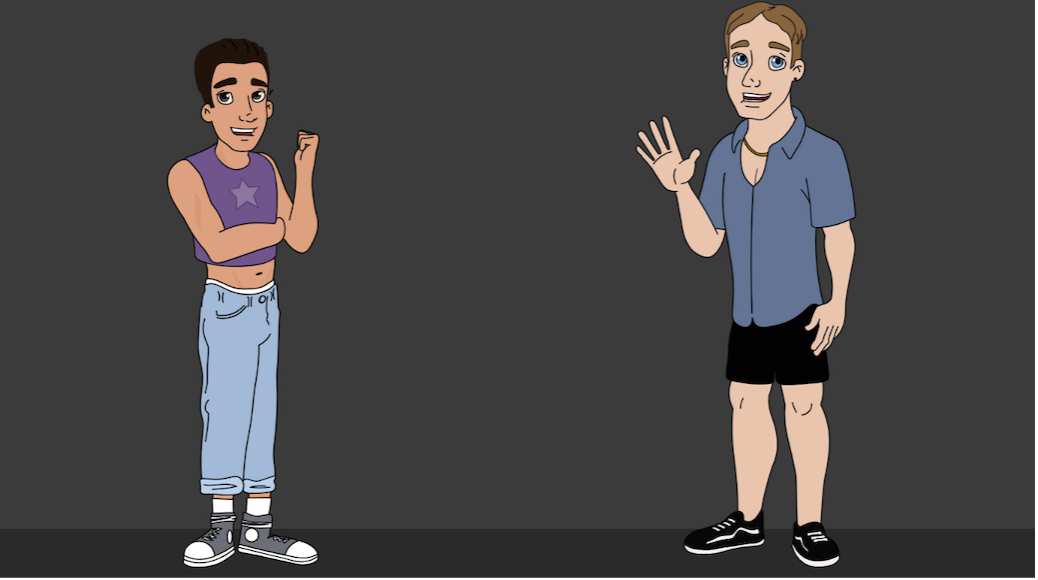
New narrators in the revised OurFutures Mental Health program: Alex and Ryan (Alex presented in the left and Ryan in the right).

Combining insights from interviews and consultation with LGBTQA+ youths, 3 new characters were created with the help of a professional graphic designer, reflecting a variety of genders, sexualities, and cultural backgrounds, whose mannerisms and clothes were inspired by contemporary media personalities. These new characters are displayed in Figures 1 and 2.

Julia is a transgender nonbinary queer person who is the love interest of Ella, a queer girl. Through normalizing Ella’s attraction to a transgender nonbinary queer person, these characters strive to normalize attraction, behavior, and feelings without self-doubt, self-stigma, and gatekeeping. Alex is a “femme slay queen of colour,” and Ryan is a “stereotypically hegemonically masculine-looking bro.” Together, this duo are the new story narrators who previously attended the same school as the other characters. This configuration was intentional to elevate voices within LGBTQA+ communities that have been historically underrepresented (eg, LGBTQA+ young people of color) to break down stigma and barriers to friendship and to demonstrate connection irrespective of gender and sexuality diversity. The appearances of these characters were inspired by popular television shows, including LGBTQA+ mainstays: the *Queer as Folk* reboot and *Heartbreak High*.

Feedback during stages 2.2 and 2.3 emphasized that LGBTQA+ young peoples’ stories in the past have largely revolved around surviving dysphoric adversity rather than celebrating the positive, euphoric, and mundane aspects of LGBTQA+ young peoples’ lives. Hence, Julia’s storyline does not feature any aspect of hardship in relation to their gender journey.

In addition to the 3 new characters, several minor modifications were made to the remaining cast of characters, as listed below:

- Revisions of characters maintained cultural diversity among the cast of characters.
- More realistic, discreet clothing options were sourced from popular influencers and television actors on social media.

#### Weekly, Manualized Program Content (Universal)

In response to feedback sourced from stages 1 to 4, the following adaptations were made to the content:

- We moved psychoeducation from being delivered by peers to being delivered by new older narrator characters. These narrators are alumni of the main storyline high school setting, retaining a youth voice and allowing peer-led SMS text messaging.
- We changed the character of Chloe to seem less regimented and formally trained in the way that she spoke about mental health and more of a compassionate peer who is a good listener. This ultimately allowed Chloe to more realistically represent a young person aged 14 years.
- By virtue of revising all dialogue and program content to be trauma-informed, all language used was sensitive to the impacts of adverse life experiences and how this might impact mental ill-health.

A comparison of the program content between the original and updated program is presented below in Table 1.

In the original OurFutures Mental Health, the teacher or school staff member responsible for program facilitation was provided with optional activities to administer to students after completing the cartoon component of each lesson. By contrast, the updated classroom activities contain skills practice and are, therefore, not described as optional but rather a key component of the intervention. Classroom activities are a mix of individual activity-style questions and class discussion and brainstorming activities facilitated by the teacher intended to reinforce the key concepts introduced in the cartoon storylines and provide an opportunity for skills practice using the cartoon characters’ scenarios. For each lesson, classroom activities are provided to teachers via the teacher portal of the website. These activities focus on applying the CBT strategies covered in that week’s lesson to the cartoon characters, allowing students to practice the skills learned while minimizing the potential for uncomfortable feelings that might arise when students discuss or reflect on their own personal examples in class. This adaptation reflects principles of trauma-aware schooling, which asserts that schools should promote a sense of safety among students [66]. In addition, this level of skills practice was expected to be sufficient for most children who do not experience elevated mental health symptoms, thus improving the relevancy of the program for these students.

**Table 1:** Comparison of weekly program content in original and updated versions of the OurFutures Mental Health program.

Lesson	Original content	Updated content
1	Identify the signs and symptoms of anxiety and depression.	Highlight the difference between symptoms of low mood or anxiety and depressive or anxiety disorders. Remove statistics on the prevalence of depressive and anxiety disorders to avoid students pathologizing normal experiences and to improve relatability. Encourage students to think of other words to describe low mood and anxiety to demonstrate these are common and normal emotions. Introduce the CBT^a^ model to understand how the characters’ thoughts, feelings, and physical sensations affect their behaviors. Show how the characters’ exposure to adversity (eg, bullying and domestic violence) affects their mental health. Introducing pronouns and information about experiences of gender and sexuality diversity
2	Identify unhelpful thoughts and practice skills to challenge these thoughts. Explore more realistic and helpful ways of thinking.	Identify negative thinking traps (eg, mind reading and catastrophizing) and learning strategies to challenge negative thoughts. Apply realistic thinking practice to Will’s and Ella’s scenarios, where Will’s scenario incorporates examples of applying self-compassion in light of adverse situation happening in his home life.
3	Identify behavioral strategies to improve mood and enhance mental health. Practice implementing these strategies through activity scheduling and stepladders.	General updates but no substantive changes to content or activities
4	Learn and practice skills for assertiveness. Practice skills for coping with intense emotions, for example, controlled breathing and emotion surfing.	Reflect that our past or current experiences affect how we perceive others’ communication and actions. For example, a previous experience of bullying can influence how we perceive others’ actions in the present. Assertiveness and other skills practice retained from original content
5	Structured problem-solving and behavioral experiments: learn ways to tackle daunting scenarios by breaking them into smaller, more manageable components, such as managing several assessments alongside part-time employment.	General updates but no substantive changes to content or activities
6	Review and help-seeking: promotes the importance of seeking help and reminds students of the support available. Review of strategies to address feelings of anxiety and low mood.	General updates but no substantive changes to content or activities

^a^CBT: cognitive behavioral therapy.

#### Optional at-Home Activities (Proportionate Universalism)

In the refined OurFutures Mental Health program, teachers are encouraged to additionally direct students to optional “at-home” activities that are available to download in the student portal of the website. In these activities, students are provided with psychoeducation focused on coping with adversity or traumatic events, seeking support, prioritizing safety, gender and sexuality diversity broadly, and support services specific to LGBTQA+ young people. These activities encouraged students to apply the strategies and coping skills learned in that week’s lesson to themselves and their own scenarios. These included activities such as grounding exercises, soothing activities, imagery, and self-compassion. Teachers were informed that these activities were to be accessed by students outside of class time to ensure that this self-practice was voluntary and confidential. Specific content is presented in Textbox 7. These opt-in at-home activities were introduced to facilitate a restructuring of the program to provide low-intensity therapeutic content to *all* students, including students not exhibiting mental ill-health symptoms, while providing greater-intensity therapeutic content to students who might need it. Therefore, aligned with the concept of proportionate universalism, these optional activities facilitated an “opt-in” increased intervention dosage for students who perceived the need or wanted additional information and skills training while retaining a low-dose, universal mental health intervention for delivery within schools.

**Summary of optional, at-home activities available to students.**

**Summary**

- Psychoeducation: apply the cognitive behavioral therapy model to self to identify the thoughts, feelings, and behaviors associated with recent feelings or low mood or anxiety and identify ways of coping with these. Identify which of these ways of coping were helpful or unhelpful. Provide psychoeducation about stressful life events, stigma and discrimination, and our environment on mental health. Identify sources of support for managing mental health and provide contact details for third-party support services.
- Cognitive strategies: apply realistic thinking and thinking traps to the student’s own personal example.
- Behavioral strategies: complete behavioral strategies for oneself, for example, stepladder and activity scheduling.
- Assertiveness training: practice assertiveness in response to a situation the student is currently facing (or using an example from the past). Introduce and practice additional strategies (than those provided in the universal content) to cope with intense emotions, for example, grounding, soothing, imagery, and self-compassion. Encourage students to select and try some of these strategies and record which ones they found helpful.
- Problem-solving: apply structured problem-solving to a problem the student is currently facing (or one from the past). Encourage students to reflect on their progress or the success of their structured problem-solving attempt after they enact the solution they came up with and try a different strategy if needed.
- Review and sources of support: review the techniques and strategies learned throughout the program. Record sources of support available to the student and encourage them to identify barriers to reaching out to this person and ways to overcome barriers to reaching out.

#### Weekly Student and Teacher Lesson Summaries

Student and teacher lesson summaries were updated to include more guidance on trauma and gender and sexuality diversity, including the relationships between traumatic events and gender or sexuality stigma and discrimination on mental health; inclusive, trauma-informed language regarding gender and sexuality; and what schools and teachers can do to support the mental health of their students, particularly LGBTQA+ students and students exposed to adversity.

#### Quiz Questions to Reinforce Material

Multiple-choice quiz questions at the end of each module were also modified to reinforce key concepts regarding problem-solving skills, coping skills, and the interconnectedness of thoughts, behaviors, and emotions (eg, “True or false: Avoiding things you are anxious about can make anxiety worse in the long term.”). In addition, some items were included to promote principles of trauma-informed approaches, particularly related to recognizing the signs, symptoms, and impacts of exposure to adversity. Finally, quiz items were included that aimed to promote students’ knowledge and acceptance of gender and sexuality diversity. The questions and answers specific to LGBTQA+ young people aimed to reinforce key skills of allyship, fostering acceptance, inclusion of LGBTQA+ young people, and affirming and validating LGBTQA+ young people. For example, 1 answer delivered by Josh is as follows:

Love is love! No matter if its cis people, hetero people, trans people, or queer people, relationships are the same for everyone.

## Discussion

### Principal Findings

The updated OurFutures Mental Health program is a trauma-informed, LBGTQA+ affirmative program aligned with the concept of proportionate universalism. Program adaptations included (1) adding new characters and storylines to reflect gender and sexuality diversity and common adverse life events, youth perspectives, and expert advice; (2) updating teacher resources to include more guidance on trauma, guidance on LGBTQA+, and teaching social-emotional skills; and (3) restructuring the content in line with targeted universalism, by providing lower-intensity social-emotional skills and psychoeducation to all adolescents in the core modules or activities and additional content via voluntary, at-home activities (eg, LGBTQA+ and trauma resources and further skills for depression or anxiety). This universal, student-facing intervention will use proven strategies to enhance implementation and scalability in schools. This includes web-based delivery of the lesson content via a web portal, student and teacher summaries, and discussion guides (not requiring teacher training). The content of the program is also mapped to the Australian National Curriculum, allowing it to be embedded in teacher planning for mental health education.

The OurFutures Mental Health program adaptation responds to recent mixed findings of universal school-based mental health prevention programs, which include null, small beneficial, and small iatrogenic effects [19,20,23,37,42]. The reality of diverse school student populations presents a challenge for preventionists to ensure the relevance and utility of universal mental health prevention programs for all students. Our findings highlight that the heterogenous distributions of the severity of mental ill-health symptoms among young people are meaningful for how relevant and engaging universal mental health prevention programs are to end users [27]. This study highlights the importance of sourcing formative insights from a variety of stakeholders, especially young people themselves. Asking what works, for whom, and why it works is of crucial importance in determining the efficacy of prevention approaches [19,20].

The efficacy of the refined OurFutures Mental Health program is currently being tested through a cluster RCT with up to 1200 students in 12 schools across Australia [44]. We hypothesize that, compared with students in control schools, students in intervention schools will show improved mental health knowledge and reduced depressive and anxiety symptoms at 3 months after baseline. A planned subgroup analysis will also examine the program effects for those exhibiting elevated mental health symptoms at baseline, across the 3 main outcomes of knowledge, depression, and anxiety symptoms. Secondary outcomes to be measured include improvements in general well-being, help-seeking, and quality of life and reductions in psychological distress; functional impairment; social, emotional, and behavioral problems; and alcohol use. In conjunction with student-report indicators of mental health and well-being, the proposed trial will also administer brief evaluation surveys to students and teachers in intervention schools to assess their experiences (eg, perceived satisfaction, relevance, helpfulness of program materials, and potential areas for improvement) completing or delivering the OurFutures Mental Health program. Teachers in intervention schools will also complete weekly logbooks describing their implementation of the program and any associated barriers and facilitators. A formal process evaluation will analyze and summarize findings from both evaluation surveys and logbooks to identify areas for improved relevance and engagement in future program iterations, with particular attention to the acceptability and reception of intervention characteristics tailored to LGBTQA+ young people and young people exposed to adversity. These results will also be compared with process evaluation results related to the previous iteration of the program [27] to further identify strengths and weaknesses for future program design and adaptation.

The adaptation process described in this study is not without its limitations. First, focus groups with grade 8 students were conducted at 3 independent or private schools in Sydney, Australia, and only 2 LGBTQA+ young people agreed to participate in targeted interviews. While this overrepresentation of youth from independent schools, who are likely from higher socioeconomic backgrounds, and urban youth limits generalizability to diverse groups of youth, qualitative responses were detailed and aligned with evidence from national surveys on sources of concern among young Australians [67]. Similarly, the limited number of individual interviews with LGBTQA+ youth may not have captured the full diversity of experiences within this population. Future studies should aim to increase the sample size and diversity of students to allow for more generalizable findings. Second, there was a poor quality of audio recordings for certain focus groups, which, in turn, may have missed participant insights during transcription. Every effort was made to transcribe these focus groups accurately with careful double-checking of transcripts for quality assurance purposes. Ensuring high-quality recording equipment should be prioritized in future studies. Third, the original evaluation data sourced for stage 1 of the adaptation process are relatively dated (having been collected in 2015). The nonrepresentativeness of this cohort relative to more contemporary cohorts was mitigated by our sourcing of feedback from additional contemporary youth (stage 2). Finally, due to time and budgetary constraints, not all updates to cartoons could be executed.

### Conclusions

To the best of our knowledge, our study is the first to represent a shift in thinking about the future of school-based, universal mental health prevention, adapting a universal mental health prevention program to be universally proportionate, thereby leveraging the strengths of both universal and targeted approaches. Specifically, our study thoroughly details the multistaged adaptation of a universal mental ill-health prevention program, OurFutures Mental Health, to be trauma-informed, LGBTQA+ affirmative, and relevant to contemporary youth, triangulating multiple sources of quantitative, qualitative, and evidence synthesis data. A school-based cluster RCT with grade 8 students in Australian secondary schools is currently underway to evaluate the effectiveness of the OurFutures Mental Health program for reducing depressive and anxiety symptoms and improving mental health knowledge. Our study proposes a novel solution to the global call to action issued to actors in the school-based mental health prevention space to decisively and innovatively respond and advance the current stalemate state of the literature to ultimately improve the mental health and well-being of young people in schools.

## Appendix

Multimedia Appendix 1 Focus group and interview guides.

## Abbreviations

CBT: cognitive behavioral therapy
LGBTQA+: lesbian, gay, bisexual, transgender, nonbinary, queer, questioning, and otherwise gender and sexuality diverse
RCT: randomized controlled trial
YAC: youth advisory committee
