# Supplementary material for: Refining the Universal, School-Based OurFutures Mental Health Program to Be Trauma Informed, Gender and Sexuality Diversity Affirmative, and Adherent to Proportionate Universalism: Mixed Methods Participatory Design Process
Source: JMIR Pediatr Parent. 2024 Aug 21;7:e54637. doi: 10.2196/54637 (PMC11375394; doi:10.2196/54637)

**Supplementary Materials**

**Appendix A – Focus Group Guide**

*Part 1: Wellbeing and mental health of young people*

1. What are some of the main concerns or stressors that bother young people today?
2. How do these stressors impact young people’s wellbeing and mental health?
3. When young people think about <stressor/s>, how does that make them feel?
4. There are sometimes good ways to handle stress or bad experiences, and sometimes not-so-good ways of coping. How do young people cope with/handle these experiences in their lives? What are some things young people might do to make them feel better/less worried/forget about it?
5. What kind of topics/issues do you think we should include in the character stories in a wellbeing program?

*Part 2: Thought-Challenging Activity*

Let’s now look at some examples of the characters and stories in our program. (Ask students to break into small groups and allocate a brief scenario (hard copy) in the OurFutures student modules to each group).

1. Ask students in their groups to think about a new character their age and describe them; think of a stressful or worrying situation they might be experiencing; describe what they are thinking, feeling, physical sensations; describe what they end up doing as a result of these thoughts and feelings (if the resulting behaviour isn’t helpful, ask the group to come up with a more helpful coping strategy).
2. After 5 minutes for review, bring students back to one group and ask them to talk through and share their thoughts, prompting with follow-up questions where relevant.

*Part 3: Review of OurFutures modules*

Draw students’ attention back to allocated scenarios/cartoons. After 5 minutes for review, ask them the following questions:

1. Do you think the content / language / scenarios are relevant to young people? Are there any young people who might not find it relevant? If so, what parts would you change?
2. Do you have any comments about the graphics and layout of the module?
3. Are there any other suggestions you have for updating the OurFutures modules – what would you add or change?
4. Before we finish, is there anything else you think would be important for us to know or consider for our program?

*Part 4: Conclusion and de-brief*

Indicate to students the focus group has ended: e.g., ‘This concludes our focus group session. Thank you everyone for being such fantastic participants, we have learned a lot and will take all of these insights and ideas back to our team.’ Proceed to ask the following de-brief questions

1. While we transition back to class, please feel free to share your thoughts about that focus group – what did you like/not like about the focus groups? What are your suggestions for improving them?
2. Provide snacks to focus group participants.

**Appendix B – Interview Guide**

The aim of this study is to hear from young people to update an online mental wellbeing program, *OurFutures: Mental Health*, we are seeking input from young people to update the program and improve inclusivity and sensitivity to a diverse range of student backgrounds and experiences. Considering the particularly challenging last few years, there is an even greater need to address the mental health and wellbeing of our young people, through programs that are relevant and inclusive.

To update the program, we will be conducting interviews with students from the rainbow community, to ensure the content and scenarios are relevant to gender and sexuality-diverse young people living in Australia today.

All information discussed will remain CONFIDENTIAL, unless you tell us you are being harmed, or at risk of being harmed, by yourself or someone else. Then we might have to tell someone to keep you safe. We will talk to you before we tell anyone what you said.

Do you have any questions before we begin?

I’ll start by asking you for some information about yourself.

Demographics:

1. What is your age?
2. How do you describe your sexual orientation?
   1. Straight (heterosexual)
   2. Gay or lesbian
   3. Bisexual
   4. I use a different term (please specify)
   5. Don’t know
   6. Prefer not to answer
3. How do you describe your gender?
   1. Man or male
   2. Woman or female
   3. Non-binary
   4. I use a different term (please specify)
   5. Prefer not to answer
4. At birth, you were recorded as:
   1. Male
   2. Female
   3. Another term (please specify)

**(**The questions below will guide the interviews. They will be asked aloud and elaboration will be encouraged).

*Part 1: Mental health*

These next questions will ask about your mental health. When I ask these you can choose if you want to answer. If you don’t want to talk about something, that’s ok. You can stop talking to us at any time if you don’t want to talk to us anymore.

1. What do you think are the main concerns that bother young people from the rainbow community today? (Encourage elaboration on concerns that are brought up)
2. (If not previously mentioned, direct youth to discuss the following) How does your gender and/or sexual orientation impact your life or wellbeing? Does it?
3. What are some of the situations or sets of events that make you feel not affirmed, supported, accepted or safe with regards to your gender and/or sexuality?
   1. Conversely, what are some of the situations or sets of events that make you feel affirmed, supported, accepted or safe with regards to your gender and/or sexuality?
4. When you think about situations or sets of events that are not accepting or safe for people of diverse genders and/or sexualities, how does that make you feel?
   1. On the other hand, how do you feel when you think about situations or events that are accepting and safe for people of diverse genders and/or sexualities?
5. How do you cope with/handle these things in your life? When you think about these things, are there any things you do to make you feel better/less worried/forget about it?
6. What kind of topics/issues related to gender and sexuality-diversity do you think we should include in the character stories in future OurFutures modules?

*Part 2: Review of OurFutures modules*

Ask youth to go through extracted example scenarios from the OurFutures Mental Health student modules. After the young person has had 5 minutes to review, discuss the following:

1. What were your first impressions? Were there any character stories or pictures that you relate to? Why? Were there any that seem outdated or irrelevant? Why?
2. Do you think the content / language / scenarios are relevant to gender and sexuality-diverse young people? If not, what would you change?
3. Do you have any comments about the graphics and layout of the module?
4. Discuss any other suggestions you have for updating the OurFutures modules – what would you add or change?

**Appendix C - Concerns, impact, ways of coping, and topics to include in mental health prevention programs as recommended by focus group participants, aged 13-15 years old.**


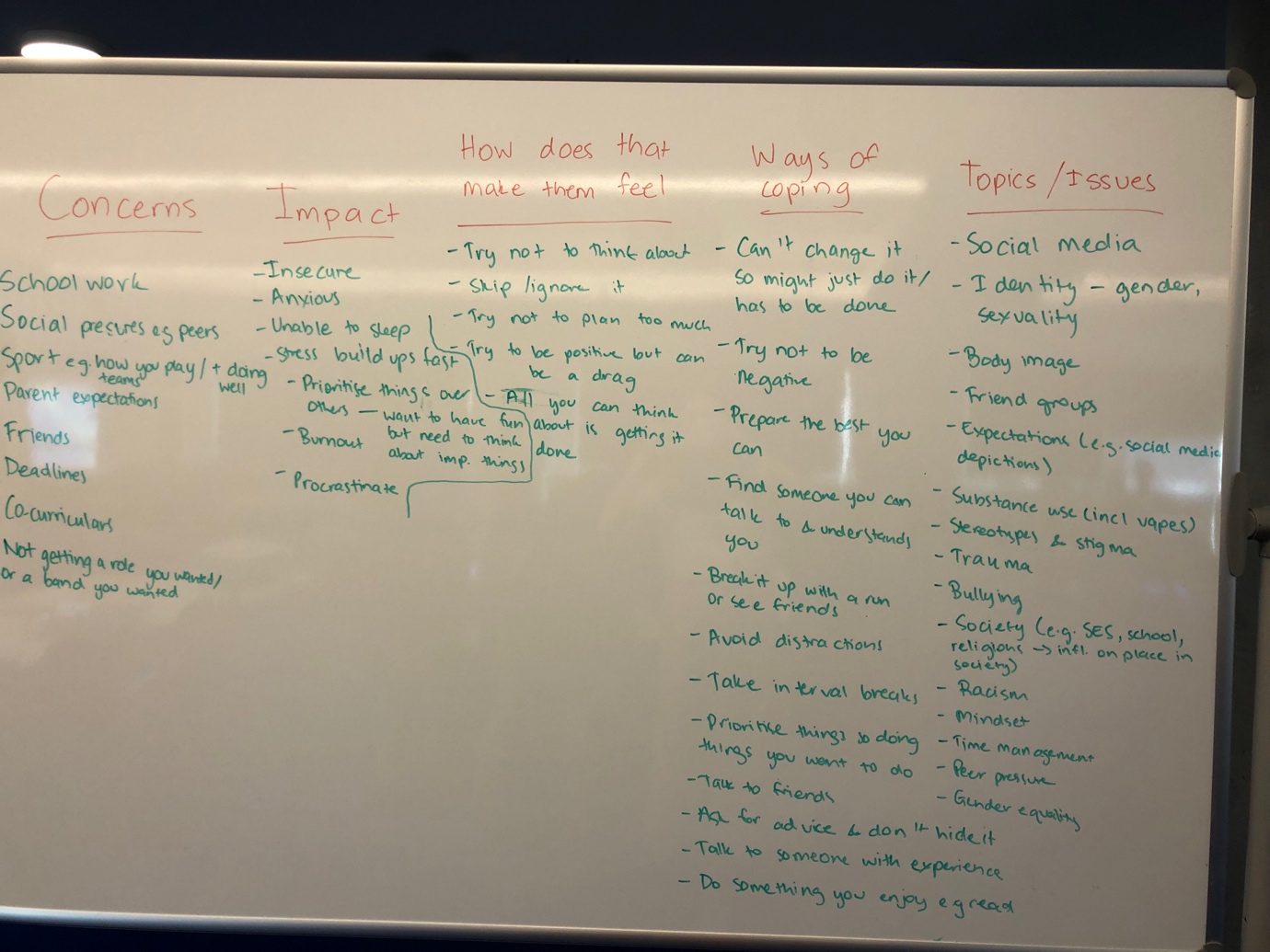

Supplement: Multimedia Appendix 1 [file pediatrics_v7i1e54637_app1.docx]
